# Study of bone remodeling of two models of femoral cementless stems by means of DEXA and finite elements

**DOI:** 10.1186/1475-925X-9-22

**Published:** 2010-05-28

**Authors:** Luis Gracia, Elena Ibarz, Sergio Puértolas, José Cegoñino, Fernando López-Prats, Juan J Panisello, Antonio Herrera

**Affiliations:** 1 Department of Mechanical Engineering, University of Zaragoza, María de Luna 3, 50018 Zaragoza, Spain; 2 Department of Orthopaedic Surgery and Traumatology, Miguel Hernández University, Crta. Nacional N-332 s/n, 03550 Sant Joan, Alicante, Spain; 3 Department of Orthopaedic Surgery and Traumatology, Hospital of Elche, Camí Almassera 11, 03203 Elche, Alicante, Spain; 4 Department of Surgery, University of Zaragoza, Domingo Miral s/n, 50009 Zaragoza, Spain; 5 Department of Orthopaedic Surgery and Traumatology, Miguel Servet University Hospital, Paseo Isabel la Católica 1, 50009 Zaragoza, Spain

## Abstract

**Background:**

A hip replacement with a cemented or cementless femoral stem produces an effect on the bone called adaptive remodelling, attributable to mechanical and biological factors. All of the cementless prostheses designs try to achieve an optimal load transfer in order to avoid stress-shielding, which produces an osteopenia.

Long-term densitometric studies taken after implanting ABG-I and ABG-II stems confirm that the changes made to the design and alloy of the ABG-II stem help produce less proximal atrophy of the femur. The simulation with FE allowed us to study the biomechanical behaviour of two stems. The aim of this study was, if possible, to correlate the biological and mechanical findings.

**Methods:**

Both models with prostheses ABG-I and II have been simulated in five different moments of time which coincide with the DEXA measurements: postoperative, 6 months, 1, 3 and 5 years, in addition to the healthy femur as the initial reference. For the complete comparative analysis of both stems, all of the possible combinations of bone mass (group I and group II of pacients in two controlled studies for ABG-I and II stems, respectively), prosthetic geometry (ABG-I and ABG-II) and stem material (Wrought Titanium or TMZF) were simulated.

**Results and Discussion:**

In both groups of bone mass an increase of stress in the area of the cancellous bone is produced, which coincides with the end of the HA coating, as a consequence of the bottleneck effect which is produced in the transmission of loads, and corresponds to Gruen zones 2 and 6, where no osteopenia can be seen in contrast to zones 1 and 7.

**Conclusions:**

In this study it is shown that the ABG-II stem is more effective than the ABG-I given that it generates higher tensional values on the bone, due to which proximal bone atrophy diminishes. This biomechanical behaviour with an improved transmission of loads confirmed by means of FE simulation corresponds to the biological findings obtained with Dual-Energy X-Ray Absorptiometry (DEXA).

## Background

The implantation of a cemented or cementless femoral stem implies an important change in the physiological load distribution. The bone reacts to the new situation, in accordance with Wolff's law, undergoing a process of adaptive remodelling [1], related to both mechanical and biological factors, being the most important the initial bone mass [2].

Long term studies of different cementless stems show a high incidence of stress-shielding, caused by the change in the distribution of loads on the femur [3-5]. The monitoring of an anatomic femoral stem with metaphyseal load-bearing and HA coating (ABG-I), that was carried out through a prospective, controlled study that included 67 patients (Group I) in the period 1994-99, has confirmed that even though the clinical results are very favourable, a high percentage of cases with stress-shielding are detected [6]. This results in a proximal atrophy which has been quantified with DEXA [7]. For that reason the stem has been redesigned (ABG-II) in an attempt to improve the proximal transfer of loads and reduce the phenomenon of stress-shielding. The main differences between both stems concern geometrical design and material. The overall lenght has been reduced by 8% and the proximal and distal diameters by 10%. The prosthesis shoulder has been modified. The material has changed from Wrought Titanium (Ti 6Al-4V) alloy to TMZF (Titanium, Molybdenum, Zirconium and Ferrous) alloy.

A similar design study was done with the ABG-II stem in the period 2000-05, with 69 patients of comparable demographic characteristics than the previous one (Group II). In both studies the surgical technique, post-operative rehabilitation program, densitometry studies and statistical analysis were identical [8]. The study confirmed less proximal atrophy, therefore one could ask if the new design has effectively improved the load transfer conditions in the proximal femur, producing less stress-shielding. The simulation with Finite Elements (FE) allows us to verify the correlation between the mechanical stimulus and the changes detected in the bone density. In order to do this, the evolution of the mechanical stimulus over a period of 5 years has been analysed, correlating the findings with the quantified Bone Mineral Density (BMD) evolution in the studies using DEXA.

**Figure 1:**
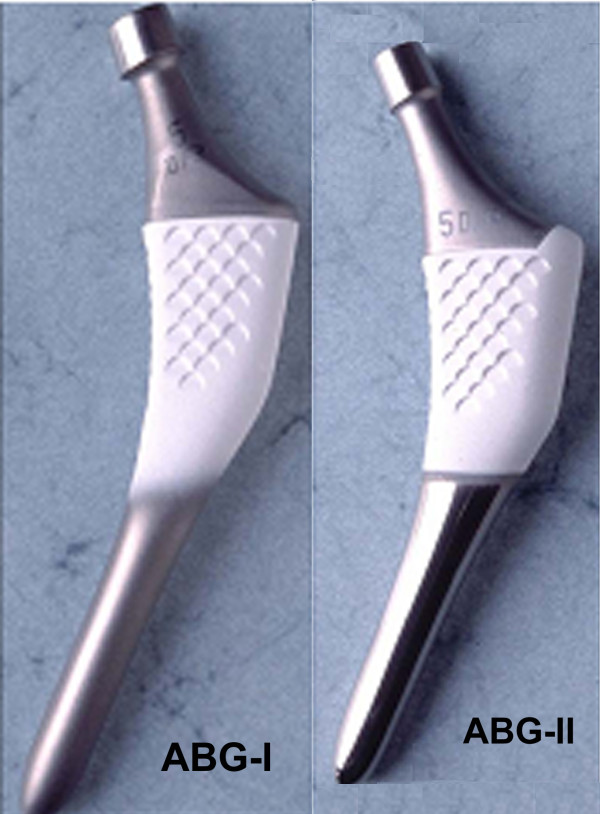
**ABG-I and ABG-II stems**.

There were several objectives to this work: firstly, to determine the long-term changes of BMD in the femur after the implantation of ABG-I and ABG-II stems (Figure 1) throughout the first five postoperative years. Secondly, to make three three-dimensional FE models of the healthy femur and femur with the ABG-I and ABG-II stem after the operation, so as to study its mechanical behaviour. This focussed on the average stresses (tension and compression) in cortical and cancellous bone for each one of the Gruen zones [9], fundamentally in what relates to the appropriate transfer of loads through contact between the bone and prosthesis. And finally, to analyse the long term differences between the implantation of an ABG-I and ABG-II prostheses to test if the changes in the design and alloy of the prosthesis produce a better transfer of loads in the proximal zone as well as its correlation with the findings of those two controlled pilot studies carried out with DEXA.

## Methods

**Figure 2:**
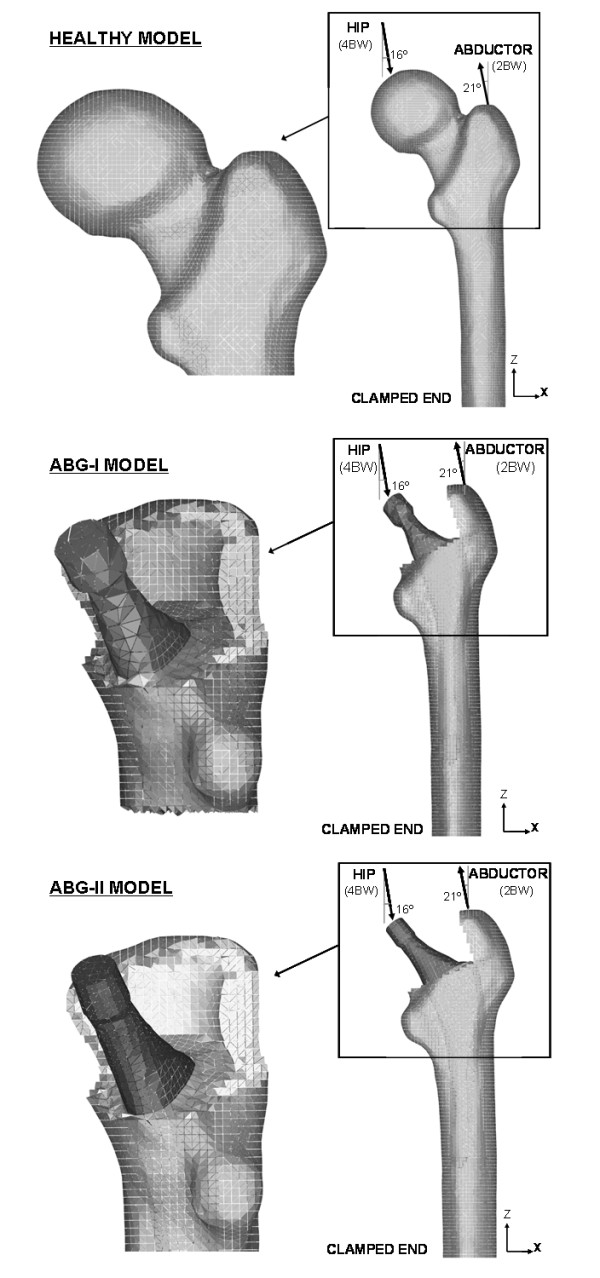
**FE models with boundary conditions of a healthy femur, the femur with prosthesis ABG-I and the femur with prosthesis ABG-II**. Detail of the mesh in the proximal area of the models.

The development of the FE models (Figure 2) was made following the same methodology as that used in [10]. For the FE simulation a cadaverous femur was used with two hip prostheses type ABG-I ad ABG-II, manufactured by Stryker. This cadaverous femur had originally belonged to a healthy 60 year old man and was only used in order to define the geometry of the model, without any relation with BMD measures. To generate the model a 3D laser scanner Roland PIZCA was used. From the scanned femur a geometric model of the outer geometry of the femur was obtained with no distinction between cortical bone, cancellous bone and bone marrow. To determine the geometry of the cancellous bone and medullar cavity 30 transverse direction (5 mm gap) tomographic cross-sections and eight longitudinal direction cross-sections were taken using CT Scan (General Electric Brightspeed Elite). A three-dimensional mesh of healthy femur, based on linear tetrahedral elements (Figure 2, healthy model), was made in I-deas software [11]. So as to develop the pattern with prosthesis, an ABG-I prosthesis was scanned to obtain its geometry. Afterwards we proceeded with the operation on a cadaver femur with a prosthesis being implanted in the same way as a real hip replacement operation would be carried out.

Once the three meshes had been generated in I-deas (healthy femur, prosthesis ABG-I and operated femur), the prosthesis was positioned in the femur always taking the mesh of the operated femur as the base. From the previous process of modelling on the cadaveric femur, only the cortical bone was used, from which the cancellous bone was modelled again, in such a way that it fit perfectly to the contact with the prosthesis (Figure 2, ABG-I model). Work with the ABG-II prosthesis was undertaken in the same way (Figure 2, ABG-II model).

The program *Abaqus 6.7 *[12] was used for the calculation, with the *Abaqus Viewer *being used for the results postprocessing. It was necessary to undertake a contact simulation between the prosthesis and the cancellous bone for which a friction coefficient of 0.5 was considered simulating the press-fit setting according with [13]. In light of the former results a sensitivity analysis was carried out in order to determine the appropriate interface conditions, considering several friction coefficient values from 0.2 to 0.5 in steps of 0.05, obtaining significative differences in the analyzed range, but with similar results from 0.4 to 0.5. It was observed that the value of 0.5 corresponds practically to a bonded interface, but with the advantage that allows moving apart the stem from the bone in higher tension zones, providing a more realistic stress distribution inside the bone.

The final model with ABG-I stem comprises a total of 60401 elements (33504 for cortical bone, 22088 for cancellous bone and 4809 for ABG-I stem). The final model with the ABG-II stem is made up of 63784 elements (33504 for cortical bone, 22730 for cancellous bone and 7550 for ABG-II stem).

Three boundary conditions were defined: clamped in the medial diaphyseal part of the femur, force on the prosthetic head due to the reaction of the hip caused by the weight of the person (400% BW) and force on the greater trochanter (200% BW) generated by the abductor muscles [10].

The values of the mechanical properties used in the prostheses as well as the biological materials are shown in Table 1. These values have been obtained from the bibliography specializing in the subject [14-22] and they have been simplified considering an isotropic behaviour. In the design of the ABG-II second generation prosthesis a different titanium alloy was used to that of the ABG-I. The prosthetic ABG-I stem is made with a Wrought Titanium (Ti 6Al-4V) alloy, the elasticity modulus of which is 110 GPa. On the other hand the TMZF alloy which is used on the ABG-II stem has a Young's modulus of 74-85 GPa, according the manufacturer information, using a mean value of 79.5 GPa in the different analyses.

**Table 1:** Mechanical properties of materials

	ELASTIC MODULUS	POISSON RATIO	MAXIMUM COMPRESSION STRESS	MAXIMUM TENSION
	(MPa)		(MPa)	(MPa)
**CORTICAL BONE**	20000 ^15, 20, 21^	0.3 ^16, 18^	150 ^16, 18^	90 ^16, 18^
**CANCELLOUS BONE**	959 ^14^	0.12 ^17^	23 ^16, 18^	
**BONE MARROW**	1 ^16, 18^	0.3 ^16, 18^		
**ABG-I STEM**	110000 ^22^	0.33 ^19^		
**ABG-II STEM**	74000 - 85000 ^22^	0.33 ^19^		

**Figure 3:**
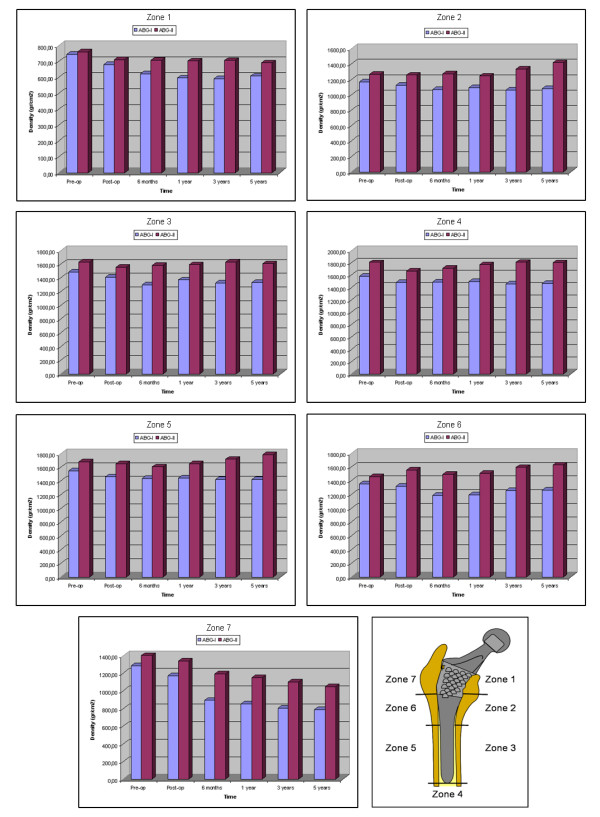
**Evolution of bone mass density for ABG I (bone mass group I) and ABG II (bone mass group II), corresponding to five years, in the Gruen zones**.

The bone density evolution in the operated and healthy hip is reported in [8]. Taking various studies as reference [23,24], a linear relationship between the bone mass values [8], which come from the medical study collected in [7], and the apparent density was established in addition to a cubic relationship between the latter and the elastic modulus, using a maximal Young's modulus of 20 GPa, thereby obtaining the cortical bone modulus of elasticity values for each one of the 7 Gruen zones (Figure 3). To carry out the analysis of the results, the cortical bone of each model is divided into seven zones which coincide with the Gruen zones. The elastic modulus obtained from the values in [8] was used as an input in the cortical bone. These values are being successively adjusted for each one of the models (femur with ABG-I stem and femur with ABG-II stem) in different moments of time: post-operative, 6 months, 1, 3 and 5 years. In addition, the initial data corresponding to the pre-operative moment are used as an input in the healthy model. The mechanical properties of the cortical bone have been calculated from the bone mass data from groups I and II respectively.

For the complete comparative analysis of both stems, all of the possible combinations of bone mass (group I, ABG-I, 67 patients in the period 1994-99 and group II, ABG-II, 69 patients in the period 2000-05) prosthetic geometry (ABG-I and ABG-II) and stem material (Wrought Titanium or TMZF) were simulated. This way it was possible to compare the mechanical performance of both prostheses in what refers to the transmission of loads and the interaction in the bone-prosthesis contact zone. It also makes it possible to distinguish the most influential parameter (geometry or material) for the design of future prosthetic stems.

The average von Mises stress is used, given that despite not distinguishing between tension and compression values, it is sufficiently indicative of the tendency of the mechanical stimulus and it is standard in FE software.

## Results

Both models with prostheses ABG-I and II have been simulated in five different moments of time which coincide with the DEXA measurements: postoperative, 6 months, 1, 3 and 5 years, in addition to the healthy femur as the initial reference. In both groups of bone mass an increase of stress in the area of the cancellous bone is produced, which coincides with the end of the HA coating, as a consequence of the bottleneck effect which is produced in the transmission of loads, and corresponds to Gruen zones 2 and 6, where no osteopenia can be seen in contrast to zones 1 and 7.

BMD evolution in the operated and healthy hip is shown in Figure 3 for both prostheses. For ABG-I, the preoperative measurements performed in both hips showed slightly higher BMD rates in the healthy hip, although these were not statistically significant. Postoperative values were taken as a reference for the operated hip. Differences between 2.66 and 10.01% in respect to those measurements taken before surgery were found and attributed to bone loss due to the surgical procedure. A decrease in BMD was detected in all zones except zone 4, six months after surgery. Between 6 and 12 postoperative months there was a slight additional loss of BMD in zones 1 and 7, but some bone recovery in the middle and distal areas around the implant. No significant changes in BMD were observed in zones 1 to 6 from the end of the first year to the end of the fifth year.

For ABG-II, the preoperative measurements performed in both hips showed again slightly higher bone density rates in the healthy hip, ranging between 2.2% in zone 5 to 4.1% in zone 2, although these were not statistically significant. Differences between 0.63 and 6.43% in respect to those measurements taken before surgery were found and attributed as in the ABG-I case to bone loss due to the surgical reaming and rasping. No changes or a minimal decrease in bone density was detected in zones 1 to 6, six months after surgery, attributed to rest period, partial weight bearing and the later effects of surgery. The bone loss was statistically significant only in zone 7. A slight additional loss of bone density was observed in zone 7, as well as some bone recovery in the middle and distal areas around the implant. Minor changes in bone density were observed in zones 1 to 6 from the end of the first year to the end of the fifth year. The bone mass remains stable in this period, with a little bone recovery in zones 2 and 6. Nevertheless, there was some decrease in zone 7 in the period between the first and fifth year, when a loss of 23.88% can be reached. The bone density in the contra-lateral healthy hip (bone mass group II) showed some slight differences during the follow-up, with decreases between 1.4 and 2.7%, more evident in the proximal part of the femur, richer in cancellous bone. The values obtained for zones 3 to 5 were similar to those of the operated femurs; in zones 2 and 6 they were slightly superior; only zones 1 and 7 showed significant differences.

**Figure 4:**
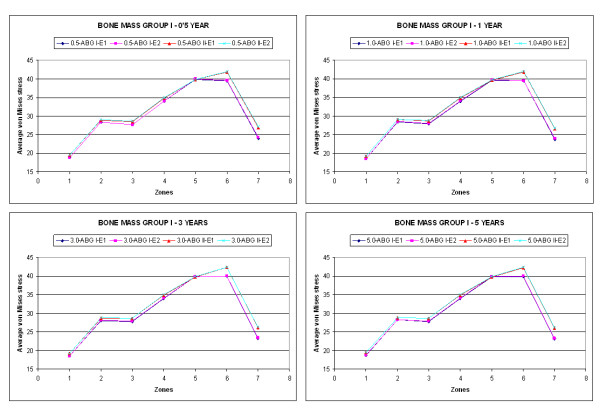
**Comparison of the average von Mises stress as a function of design and material for bone mass group I**.

**Figure 5:**
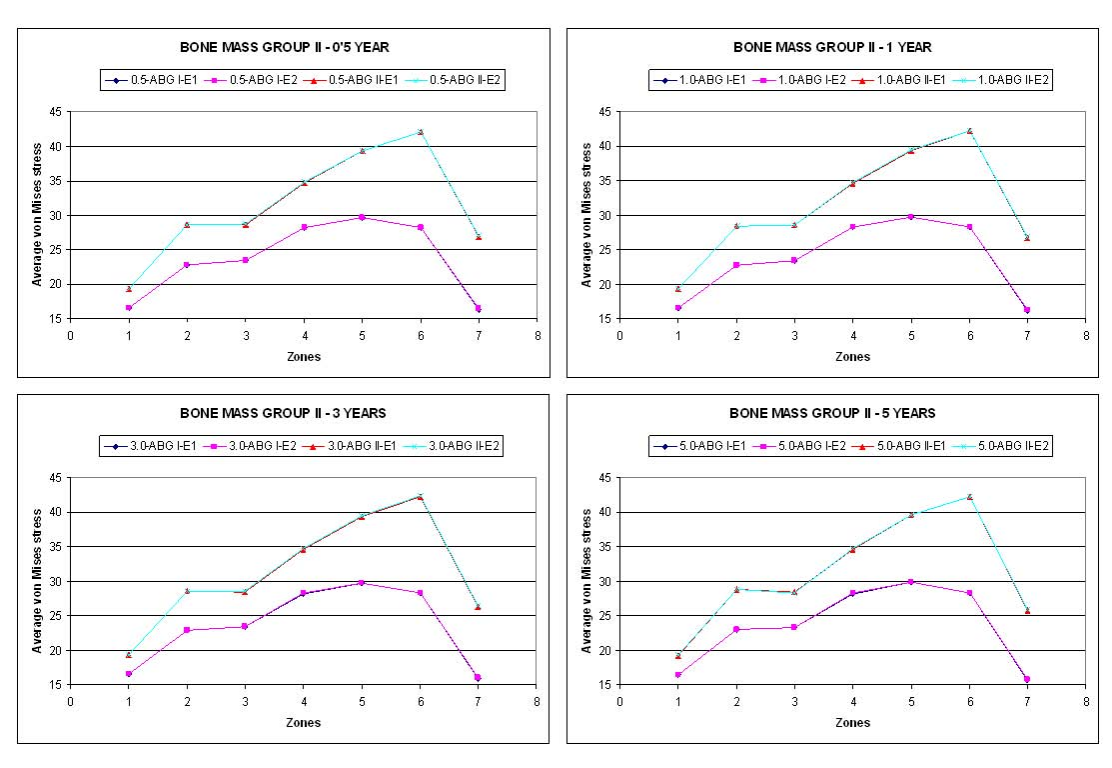
**Comparison of the average von Mises stress as a function of design and material for bone mass group II**.

Figure 4 shows the results of the average von Mises stress (MPa), corresponding to the combinations of geometry (ABG-I, ABG-II) and prosthesis material for group I of bone mass at five years, and Figure 5 shows the equivalent results for group II of bone mass. It can be confirmed that the global behaviour of the prostheses is the same in both models; however, in the case of the ABG-II stem higher stress values are reached on both the cancellous and cortical bones, fundamentally in the proximal zones.

A tensional increase is noticeable in the whole area close to the lesser trochanter with the use of the ABG-II stem, as well as the tensional increase that the insertion of the ABG-II prosthesis involves with respect to the ABG-I. These differences can be observed in a more clear way in Figures 4 and 5. In both figures it is clearly noticeable that the result corresponding to the two material for every stem are practically the same (superimposed lines); however, the results corresponding to both geometrical designs (ABG I, ABG II) are different, with a higher tensional level for the ABG II stem.

It could be checked that in every case the stress corresponding to the ABG-II stem is greater than the one resulting from the insertion of the ABG-I stem (Figures 4 and 5). In the figures it can be seen that in every zone and for any time the stress achieve higher values in ABG-II than in ABG-I stem. This way it is possible to confirm that with the second generation of stem (ABG-II) the stress increases in practically every zone with this increase being most evident in zone 7.

**Figure 6:**
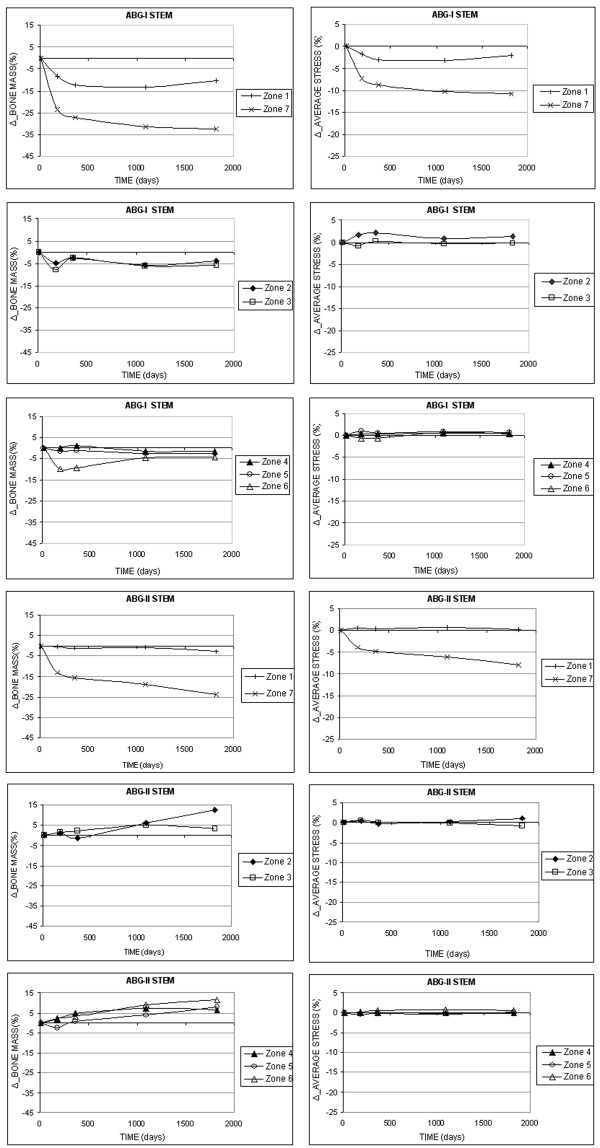
**Bone mass (%) variation versus time and variation in average von Mises (%) stress versus time for the femur with prosthesis ABG-I and ABG-II in the Gruen zones**.

Figure 6 shows the evolution of the bone mass (%) and the average von Mises stress (%) for each one of the 7 Gruen zones in both models, considering the corresponding group and material for each stem.

## Discussion

When a hip arthroplasty is carried out the initial tensional state of the joint is modified. In the healthy femur, the loads are transferred from the femoral head to the lesser trochanter, which distributes the compressive forces to the femoral diaphysis [25]. This way the primary trabecular bundles on the healthy femur are identified: the arch shape, formed by tension forces and the principal compression group of Delbet, formed by compression forces [26]. This model of loads is inverted after the implant. It is confirmed that the stresses are transferred fundamentally from the prosthetic head to its stem, producing stress-shielding and loading the femur mainly in the areas which correspond with the end of the HA coating (Figure 4 and 5).

The simulation using FE makes it possible to explain the biomechanical changes produced in the femur after the implant of a prosthetic stem and establish an evident parallelism between the results obtained in the study with DEXA and the results from the study with FE, as can be seen in Figure 6. The bone mass variation and the tensional evolution in each one of the Gruen zones obtained by simulation follow similar paths.

An essential point in the models concerns the loads. The most significant correspond to the gluteus medium muscle, iliotibial tract and psoas major muscle or solely the action of the abductor muscles. For this study the final option was chosen in accordance with the majority of authors [23,27]. Different authors have studied the biomechanics of the hip from the point of view of contact and muscle forces [28-30]. So, according to Bergmann [30], the peak force is 330% of body weight (BW) when going downstairs, nevertheless he accepts that higher peaks could be observed (409% of BW during walking) when muscle dysfunction exists [29].

If the principal stress flows (tension/compression) are represented in the models with stem, it is possible to verify how the zone subjected to tension is found on the lateral surface of the femur, and the zone subjected to compression is on the internal face of the femur due to the eccentricity of the load. It is also possible to appreciate how the load is transferred from the prosthetic head to the femur, favouring a greater concentration of loads on the implanted bone with the ABG-II stem as opposed to the one implanted with the ABG-I stem. HA coating has no influence in BMD maintenace and evolution. Its only purpose is facilitating the osseointegration and providing a better press-fit in the metaphyseal zone [31].

In the analysis carried out with respect to material, it has been shown that the use of a new Titanium TMZF alloy does not produce any significant changes in respect to the alloy used in the ABG-I prosthesis. The reason for this is due to the fact that in both cases, the modulus of elasticity is three orders of magnitude greater than that of the modulus of elasticity of the cancellous bone, which is the bone in contact with the stem, for which the mentioned variation in the elastic modulus of the prosthesis is insufficient to provide noticeable changes. In short, the results are much more sensitive to the geometric design than to the material properties.

The results show that the ABG-II prosthetic stem is more effective than the ABG-I given that it generates higher tensional values on the bone (Figures 4 and 5), due to which the proximal bone atrophy diminishes. This biomechanical improvement with a better transmission of loads corresponds with the biological findings obtained with DEXA. We agree with [32] that a greater conservation of the proximal bone is an important biological factor, which brings about less bone atrophy in the long term. In accordance with [27], we agree that the discreet reduction in the length of the stem does not have any influence on the transmission of loads; however, it requires a more refined surgical technique to avoid the varus positioning.

Studies with DEXA in a longer term (10 years) should be needed to determine the influence of stress-shielding on adaptive bone remodelling in long term. Since this work reproduces the mechanical behaviour of two femoral stems placed in a neutral position in the femur, further studies should be addressed to determine the mechanical behaviour of the stems placed in varus or valgus positions.

## Conclusions

The study of both groups using DEXA at 5 years shows less proximal bone atrophy in the ABG-II Group, with an 8.7% improvement of the BMD [7,8]. Therefore this stem improves the proximal transmission of loads. It has been proved that the stem's smaller proximal and distal diameters, in relation to the ABG-I, conserves a greater quantity of metaphyseal bone, taking into account that it is the biological factor which has most influence in the remodelling. Concerning the changes in the design, geometric modificaciton have proved to be more effective than material replacement.

## Competing interests

The authors declare that they have no competing interests.

## Authors' contributions

LG, EI, SP and JC carried out the finite element simulations. FLP, JJP and AH carried out the DEXA studies. All authors were involved in the study design, comparative analysis between DEXA and FE simulation and writing of the manuscript. All authors read and approved the final manuscript.
